# The odour of an unfamiliar stressed or relaxed person affects dogs’ responses to a cognitive bias test

**DOI:** 10.1038/s41598-024-66147-1

**Published:** 2024-07-22

**Authors:** Z. Parr-Cortes, C. T. Müller, L. Talas, M. Mendl, C. Guest, N. J. Rooney

**Affiliations:** 1 Bristol Veterinary School, University of Bristol, Bristol, BS40 5DU UK; 2 School of Biosciences, Cardiff University, Cardiff, CF10 3AX UK; 3 Medical Detection Dogs, Milton Keynes, MK17 0NP UK

**Keywords:** Dogs, Stress, Human chemosignals, Emotions, Animal cognition, Animal behaviour, Emotion, Stress and resilience

## Abstract

Dogs can discriminate stressed from non-stressed human odour samples, but the effect on their cognition is unstudied. Using a cognitive bias task, we tested how human odours affect dogs’ likelihood of approaching a food bowl placed at three ambiguous locations (“near-positive”, “middle” and “near-negative”) between trained “positive” (rewarded) and “negative” (unrewarded) locations. Using odour samples collected from three unfamiliar volunteers during stressful and relaxing activities, we tested eighteen dogs under three conditions: no odour, stress odour and relaxed odour, with the order of test odours counterbalanced across dogs. When exposed to stress odour during session three, dogs were significantly less likely to approach a bowl placed at one of the three ambiguous locations (near-negative) compared to no odour, indicating possible risk-reduction behaviours in response to the smell of human stress. Dogs’ learning of trained positive and negative locations improved with repeated testing and was significant between sessions two and three only when exposed to stress odour during session three, suggesting odour influenced learning. This is the first study to show that without visual or auditory cues, olfactory cues of human stress may affect dogs’ cognition and learning, which, if true, could have important consequences for dog welfare and working performance.

## Introduction

The term “emotional contagion” describes how individuals in a group perceive and mimic the emotions of others around them^1^. This phenomenon is thought to be automatic and involves the processing of multiple cues, including visual (e.g. body language or expressions), auditory (e.g. voice tone, volume or pitch) and olfactory^2^. The olfactory chemo-signals released by humans during stress and fear have been shown to induce anxiety^3^ and subconsciously affect automatic defensive behaviours^4^, cognition^5^, decision-making^6^ and perception of neutral or ambiguous cues (judgment bias)^7^ in humans. These findings indicate that olfactory detection of negative emotional states in others results in more cautious or pessimistic responses, suggesting a stress “contagion” effect between humans.

Genomic and archaeological findings suggest humans and dogs have a long evolutionary history of cohabitation^8–10^. Nowadays, dogs are considered one of our closest companions^11–13^. This close relationship over tens of thousands of years has led to dogs learning to read and respond to many verbal and non-verbal cues from humans^14^. Dogs follow human pointing gestures^15,16^, gaze direction^17,18^, can learn hundreds of words^19^ and respond to different vocal tones^20^. Dogs spend longer gazing at human facial expressions accompanied by congruent than by incongruent vocalisations^21^ and show avoidance of expressions of anger and increased attention to expressions of fear^22^, suggesting an understanding of how different human emotions should look and sound.

Animals have evolved the ability to use olfactory cues to identify conspecifics as potential mates or threats^23–25^, and also as a way to signal stress^26–29^. Interspecies olfactory communication has also been demonstrated to play a role in the detection of predator species^30,31^ and familiar humans by domesticated species such as horses^32,33^ and dogs^34–36^. The link between odours and emotions in humans is strong^37^, and it is likely that dogs, with a far superior olfactory processing network^38^, also experience emotional responses to odours^39^. Indeed, there is evidence that dogs show an increase in stress-related behaviours and heart rate when exposed to odours from humans reporting fear and show an increase in stranger-oriented behaviours and a decrease in owner-oriented behaviours when exposed to odours from happy humans^40^. Dogs also exhibit a right nostril preference when sniffing human adrenaline, veterinarian sweat^41^ and the odour of a stressed dog^34^. Since olfactory signals from the right nostril are processed in the right hemisphere, which is hypothesised to be involved in the processing of negative emotions, this, too, suggests dogs perceive these odours as threatening or arousing^42^.

Studies looking at the odour signatures of stressed compared to relaxed humans show significant differences in volatile organic compounds (VOCs) released during these two emotional states. For example, VOCs in skin, sweat and breath samples collected during stress tests and relaxing activities have been shown to be significantly different within individuals^43–45^. There is evidence that dogs may pick up on these measurable differences in olfactory signals released during human stress. A study by Wilson et al. found that dogs can discriminate between stressed and non-stressed odour samples collected from human participants before and after a mental arithmetic test^46^. However, whether dogs interpreted these odours as positive or negative was not examined. The perception of stress in humans carries potential welfare implications if it negatively affects dogs’ emotional state and is an important consideration for dogs living and working in environments where human stress is high. Although dogs seem to identify the odour of human stress, its effect on their emotional state and cognitive bias has not yet been studied. We know, however, that humans show risk aversion when stressed^47–49^, and certain human chemosignals may cause people to show risk-reduction behaviours^6^. In dogs, being firmly told not to retrieve a food reward while being watched by a human, causes them to approach the reward less often and more indirectly than when the human is not watching them^50^. This suggests that dogs show risk-reduction behaviours if they anticipate a negative outcome. Since stress odours from humans are likely to be present in situations where the environment is “unsafe” or unpredictable, we predict that the odour of human stress could lead to similar risk-reduction behaviours in dogs, anticipating a negative outcome.

Cognitive or judgement bias testing is used to assess emotional states in animals based on theory and empirical findings that individuals in negative emotional states make more negative judgements about ambiguous stimuli (“pessimistic” choices) whilst those in positive states make more “optimistic” ones^49,51,52^. A more pessimistic response in cognitive bias tests has been associated with negative states in humans^47^, rats^51,53^, cows^54,55^, sheep^56^ and dogs^57^. The cognitive bias test for dogs was first described by Mendl et al. and is a spatial learning task that uses the position of food bowls as cues for relatively positive (food) and negative (no food) outcomes. It tests dogs’ responses to ambiguously located bowls as an indication of their relative pessimism or optimism and, hence, by inference, of their affective state^57^. Most studies assessing cognitive bias in dogs look for differences in cognitive bias between individuals with different traits such as personality^58^, paw preference^59^, those trained in different ways^60,61^, or with chronic conditions such as neurological disorders^62^ or separation-related problems^57^. A few studies have used the cognitive bias test to assess the effect of treatments or activities pre- and post-intervention, for example, Karagiannis et al. found dogs with separation-related problems were “less pessimistic” following treatment with fluoxetine^63^ and Duraton et al. found dogs were “more optimistic” following 2 weeks of daily olfactory-based activities^64^. Fewer studies have assessed the effect of an intervention on emotional state in the short-term, i.e. delivered *within* a session. One example is a study by Uccheddu et al., which found that exposing dogs to a blend of essential oils through an impregnated collar resulted in a “more optimistic” response compared to baseline (no odour), suggesting olfactory enrichment may acutely improve dogs’ emotional state^65^.

In the present study, we tested the effect of human stress and relaxed odours on eighteen dogs’ responses to a cognitive bias test. We hypothesised that when exposed to the odour of human stress, dogs experience a negative emotional state and take longer to approach bowls at ambiguous locations than when exposed to the relaxed odour or no odour. This study aims to better understand the role of olfaction in stress-signalling between humans and dogs and the implications on the welfare and working performance of dogs.

## Materials and methods

### Ethical approval

Ethical approval for human participants was granted by the NHS Health Research Authority (HRA) South-West Central Bristol Research Ethics Committee (REC) (reference: 22/SW/0134). All experiments were conducted in accordance with NHS HRA guidelines and regulations and followed the study protocol approved by the REC. Biological samples were collected, stored, and disposed of in accordance with the Human Tissue Act. Animal protocols were approved by the University of Bristol Animal Welfare and Ethical Review Body (AWERB) (reference: UIN-22-0102) and conducted in accordance with the UK Animal (Scientific Procedures) Act 1986. This study is reported in accordance with ARRIVE guidelines. All human volunteers and dog owners were informed about their right to withdraw, and informed consent was obtained prior to their participation.

## Experimental design

**Figure 1:**
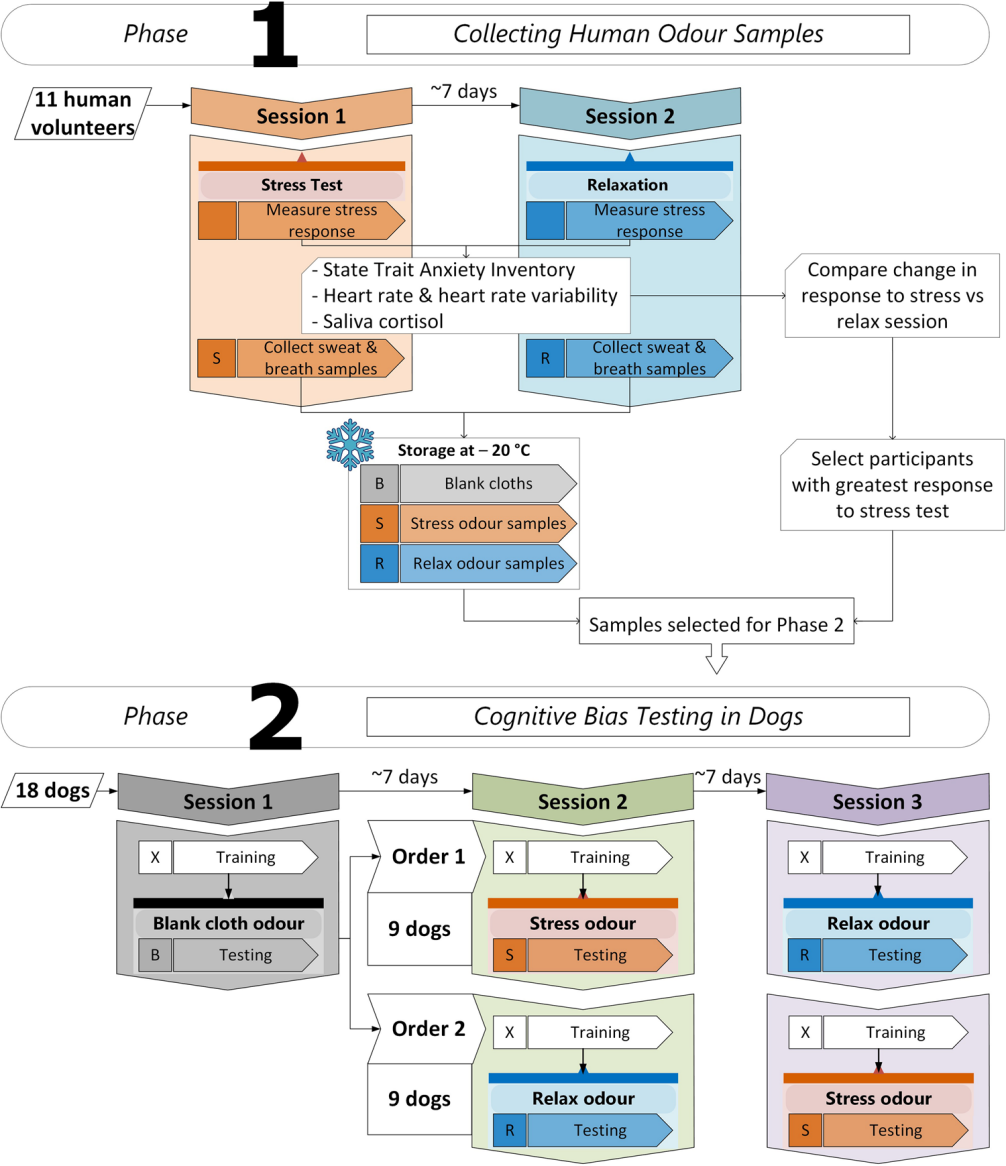
Overview of experimental design. Coloured boxes and letters indicate the odour type collected from volunteers (phase 1) or presented to dogs (phase 2): “S” (orange) = stress odour, “R” (blue) = relax odour, “B” (grey) = blank cloth odour, “X” = no odour (closed jar during training). The order of odour exposure in phase 2 was counterbalanced across all 18 dogs: Order 1 = stress odour in session 2 and relax odour in session 3, Order 2 = relax odour in session 2 and stress odour in session 3.

This study was split into two phases (Fig. 1):

1. Collecting human odour samples.
2. Cognitive bias testing in dogs.

### Phase 1—collecting human odour samples

#### Sample donors

A total of 11 volunteers (10 women and 1 man) aged 18–26 were recruited through advertisements in bulletins, notice boards and group meeting announcements at the University of Bristol Vet School. Participants were in good health, were not on any medication, had no current or prior psychiatric diagnoses, reported no recent difficulty with sleep or significant stressful events and tested negative for SARS-CoV-2 virus on lateral flow on each day of testing. Participants were instructed to abstain from alcohol and smoking for at least 12 h and from eating for 1 h before testing and to avoid spicy foods, intense exercise, using body wash, perfume, deodorants, or antiperspirants on each day of testing. Participants were informed the study involved a psychological stress test designed to induce feelings of anxiety and were advised they could stop participating at any time. Participants were compensated financially for their time and provided with snacks and refreshments at the end of each session.

#### Protocol

**Figure 2:**
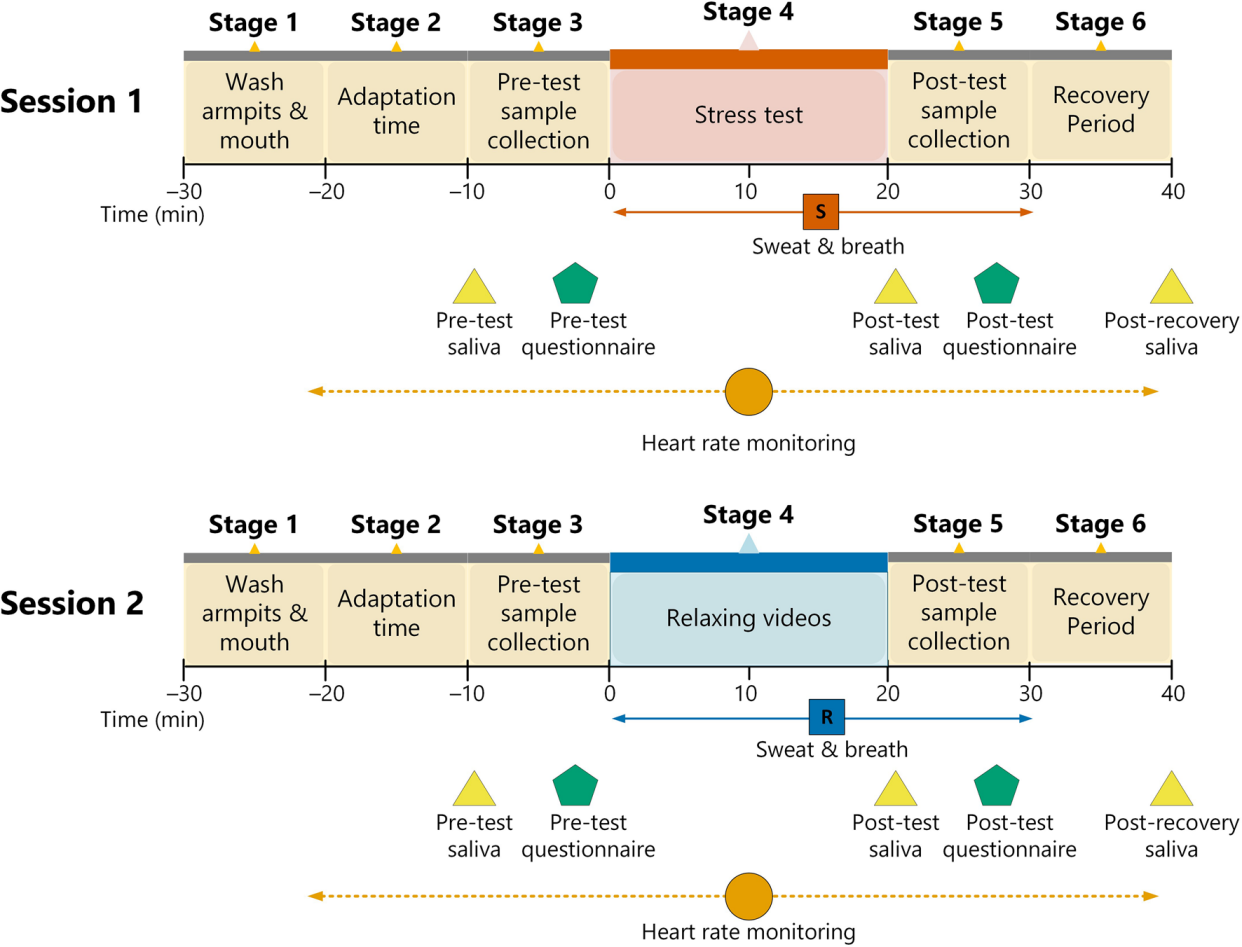
Timeline showing the protocol for session 1 (stress) and session 2 (relax) for phase 1, held 1–7 days apart. A heart monitor was attached during stage 1, and used to record heart rate and heart rate variability (HRV) during stages 2–6. A pre-test saliva sample and anxiety questionnaire were collected during stage 3 prior to the stress test/relaxation. Odour collection cloths were attached at the end of stage 3 and sweat and breath samples were collected over stages 4–5 during the stress test (“S” = stress odour) or relaxation (“R” = relax odour). A post-test saliva sample and anxiety questionnaire were collected during stage 5. A final post-recovery saliva sample was collected at the end of stage 6.

Participants attended two 1-hour long sessions held 1–7 days apart (Fig. 1). The protocol was the same on both days except for the 20-min test period, during which participants were given either the trier social stress test (TSST)^66^, previously shown to induce psychological and physiological stress^45,67–69^ or watched a compilation of forest and seaside videos shown to induce relaxation^70^. Each session had 6 stages (Fig. 2):

- *Stage 1* A heart rate monitor was attached. The participant rinsed their mouth and armpits with water prior to sample collection.
- *Stage 2* The participant sat quietly in the waiting room for 10 min.
- *Stage 3* A pre-test saliva sample and anxiety questionnaire were collected, and odour collection cloths were attached to each armpit.
- *Stage 4* The participant was called into the testing room for the 20-min stress test (session 1) or relaxing activity (session 2).
- *Stage 5* A post-test saliva sample and anxiety questionnaire were collected before the participant returned to the waiting room. Odour collection cloths were removed from the armpits, and the participant collected breath onto the same cloths.
- *Stage 6* The participant sat quietly in the waiting room for 10 min then collected a post-recovery saliva sample and removed the heart rate monitor before leaving.

All participants received the stress condition in session 1, but they were not informed of this until the test began. Once the stress test was complete, participants were informed that session 2, held 1–7 days later, would be the relaxed condition. This order allowed us to control any influence of uncertainty and anticipatory stress across the cohort, thereby maximising stress in session 1 and minimising stress in session 2. To minimise any effect of the environment reminding participants in session 2 of their previous stressful experience in session 1, the room layout was changed between sessions. To do this, the room was partitioned with a screen into two smaller spaces, with one space used for the stress test and the other for the relaxing activity. The space in which the stress test took place was, therefore, not visible to participants during the relaxing activity, minimising any visual reminders of the previous session. Following each session, participants were asked not to disclose their experience with anyone outside of the research team, as this could exclude others from participating.

#### Stress test

During session 1 (Fig. 2), the 20-min TSST (stage 4) was based on the protocol by Birkett et al*.*^66^. In brief, the primary researcher, wearing a white lab coat, called the participant into the testing room, where a second unexpected researcher was introduced as an expert in public speaking, there to assess the participant’s performance. The participant was told they had 10 min to mentally prepare a 5-min speech describing why they would be a good candidate for their ideal job and that a video of their speech would be recorded for assessment. A timer visible to the participant was set and both researchers left the room. After 10 min, the researchers returned, and the participant was instructed to deliver their speech and told they should speak for the entire 5 min. The researcher pretended to turn on the video camera and the timer was reset. If the participant stopped talking, they were prompted with the following: “You still have time remaining”. Both researchers maintained serious expressions throughout to increase social anxiety. After the 5-min speech task, the participant was instructed to subtract the number 13 from 1022 and continue subtracting 13 from the remainder until the 5 min were up, verbally reporting their answers aloud. If the participant made a mistake, they were prompted with the following: “That is incorrect; please start over from 1022”. After the 5-min maths task, the participant was informed that the test had ended and that the camera had not been recording.

#### Relaxing activity

For the relaxed condition in session 2, the primary researcher (in plain clothes) called the participant into the testing room, where a bean bag and cushions were provided for their comfort. Blankets and throws were used to cover the existing furniture and the room lighting was kept dim to create a more relaxing atmosphere. The participant was given a laptop and headphones and instructed to watch a 20-min video compilation of forest and seaside scenes with natural soundscapes. The researcher left the room and returned once the video ended.

#### Measuring stress response

To control for diurnal variations in cortisol, sessions 1 and 2 were held at the same time of day (± 2 h) for each participant. To minimise changes in saliva pH that could interfere with cortisol measurement, participants were instructed not to eat or drink (other than water) for an hour prior to each session. Each participant collected a pre-test (used as a baseline), a post-test and a post-recovery saliva sample (to measure delayed cortisol responses) (Fig. 2). Saliva samples were collected in 2 mL cryovials via passive drool and stored at − 20 °C within 30 min of collection for up to 1 month. Each saliva sample was analysed in duplicate using a Salimetrics Cortisol Enzyme Immunoassay Kit according to the manufacturer’s instructions. The assay was read using a Thermo Scientific Multiskan FC plate reader and cortisol concentrations were determined using a dose–response curve fitted in GraphPad Prism 9. Assay controls were used to confirm that the ELISA had run correctly and sample concentrations were checked against the expected range provided by Salimetrics. Heart rate and heart rate variability (HRV) were recorded using a Polar H10 chest strap with data recorded in real-time via Bluetooth onto a tablet using the Polar Sensor Logger app^71^. The primary researcher logged the start and end times for each stage of the session in real time. Data were imported into Kubios HRV software^72^ where the mean heart rate, root mean square of the successive differences (RMSSD), and Stress Index (a geometric measure of sympathetic versus parasympathetic inputs^73^) were calculated for stages 2, 4 and 6 of each session (Fig. 2). To assess subjective stress levels, participants completed a pre-test and post-test State-Trait Anxiety Inventory (STAI)^74^ for each session (Fig. 2). State anxiety scores were used to assess within-subject responses to each test condition.

#### Odour samples

Prior to entering the testing room, participants attached two 20 × 20 cm 100% cotton cloths to each armpit using micropore tape so that the cloths were in direct contact with the skin. The cloths remained in place for the duration of stages 4 and 5 (Fig. 2), after which they were removed. Participants cut each 20 × 20 cm cloth into four 10 × 10 cm pieces and exhaled a full breath onto each piece before sealing each in a separate specimen bag. This gave a total of eight pieces of cloth (four per armpit) for each donor for each session: six for cognitive bias testing of dogs and two for odour analysis (not described here). All samples were double bagged and placed on ice before being stored at − 20 °C within 30 min of collection. Samples were stored for 3–6 months until use in the cognitive bias testing (phase 2). Blank cloths used in session 1 were stored in the same conditions and for the same time as odour samples to control for “background” odour from the freezing and storage process. To maintain a within-subject design in phase 2, each dog was presented with stress and relax odours from the same donor. Since each donor collected six samples for cognitive bias testing, a maximum of six dogs could be presented with odours from the same person (without the reuse of samples). We selected three donors with the greatest stress responses across all measures during the stress test and no stress response during the relax condition (see results), which provided samples for 18 dogs.

### Phase 2—cognitive bias testing in dogs

#### Recruitment

A total of 26 dog-owner partnerships were recruited via advertisements in bulletins, posters and social media shared with staff, students, and members of the public at the University of Bristol Vet School and Langford Vets Small Animal Practice. All dogs were over 6 months of age, in good health, comfortable learning new tasks and had no history of fear or anxiety of novel environments or people. After expressing an interest, owners were invited to attend the first of three 40–60-min sessions (Fig. 1). Before starting, owners were given the opportunity to ask any questions and signed a consent form. Dogs were allowed 15 min to habituate to the room and equipment, during which the following information about the dog was collected from their owner: age, sex, neuter status, breed, whether they were registered as a teaching dog at the University of Bristol, how long they had lived with their owner, whom they were most attached to (attachment type: person present, another person, multiple people or no attachment) and how sensitive they were to their owner’s emotions (dog emotional sensitivity: scale of 1–5 where 1 = not at all and 5 = very in tune). Following the collection of this information, the first cognitive bias test began in the presence of a blank cloth (no odour). Dogs then participated in two subsequent sessions during which the stress and relax odours were introduced (Fig. 1). At the start of each session, owners confirmed their dog had been well in the preceding seven days and were asked to report any recent events that may have impacted their dog positively or negatively or any recent changes to routine. Because of the potential influence of the owner’s stress on their dog, owners (acting as handlers) were also asked to score how relaxed they felt at the start of each session (handler relaxation score: scale of 1–10 where 1 = not at all relaxed and 10 = very relaxed). Sessions were held at the same time of day for each dog approximately one week apart (6–9 days), except for on two occasions: one dog’s second session was delayed by 8 days due to the owner’s illness, and another dog’s third session was delayed by 24 days due to dog illness. Access to a water bowl was provided throughout, and dogs were never separated from their owner. All dogs that completed the study received a toy reward at the end of their third session.

#### Dogs

Of the 26 dogs recruited, eight were excluded during session 1: one dog was excluded due to mobility issues and signs of discomfort during training, two were excluded for behavioural reasons (one for showing signs of distress and the other for showing destructive behaviour towards the equipment), four dogs were excluded for failing to engage with the task for five consecutive trials and one dog due to distractibility. All 18 dogs that completed session 1 completed all three sessions of the study. These were 11 male (10 neutered and 1 entire) and 7 female (4 neutered and 3 entire) dogs ranging from 8 months to 10 years old (mean = 4.73 ± 2.67 years). Breeds consisted of 2 Springer spaniels, 2 Cocker spaniels, 2 Labrador Retrievers, 2 Braque d’Auvergne, 1 Whippet, 1 Golden retriever, 1 Miniature poodle and 7 mixed breed dogs. Eight dogs were registered as teaching dogs at the University of Bristol. These dogs will have been familiar with the study site since they often attend the veterinary school for teaching purposes (e.g. dog handling practicals). However, all dogs that participated in this study were naïve to the cognitive bias testing protocol. To control for any influence of familiarity with the study site, these eight dogs were split equally between groups and differences between teaching and non-teaching dogs were explored during data analysis (see Supplementary Analysis in Supplementary Information).

All dogs were presented with a blank cloth during session 1, after which they were divided pseudo-randomly into three groups of six, balancing for whether they were registered teaching dogs or not. Each group was subsequently presented with odour samples from one donor (Supplementary Table S1). Within each group, the order of stress and relax odour presentation was counterbalanced across dogs, so three dogs were exposed to stress odour before relaxed odour (order 1) and the other three were exposed to relaxed odour before stress odour (order 2) (Fig. 1) (Supplementary Table S1). We estimated a minimum of 18 dogs (three per group) as a realistic number of recruits in the time provided for this study. This number was based on four similar studies testing cognitive bias in dogs that used between 12 and 24 dogs (mean = 17.75)^57,63,64,75^. We were also limited by the number of donors showing a consistent stress response in phase 1 and by the number of samples collected per donor (six). We used a within-subject design to increase the statistical power; however, because the effect size and variability were unknown, a power calculation could not be performed. To maintain blinding, specimen bags containing odour samples were labelled with a code known only to a second researcher, not present during dog testing. A list of sample codes for each dog was provided to the primary researcher so that the odour type during testing was unknown to anyone present in the room.

#### Cognitive bias testing

**Figure 3:**
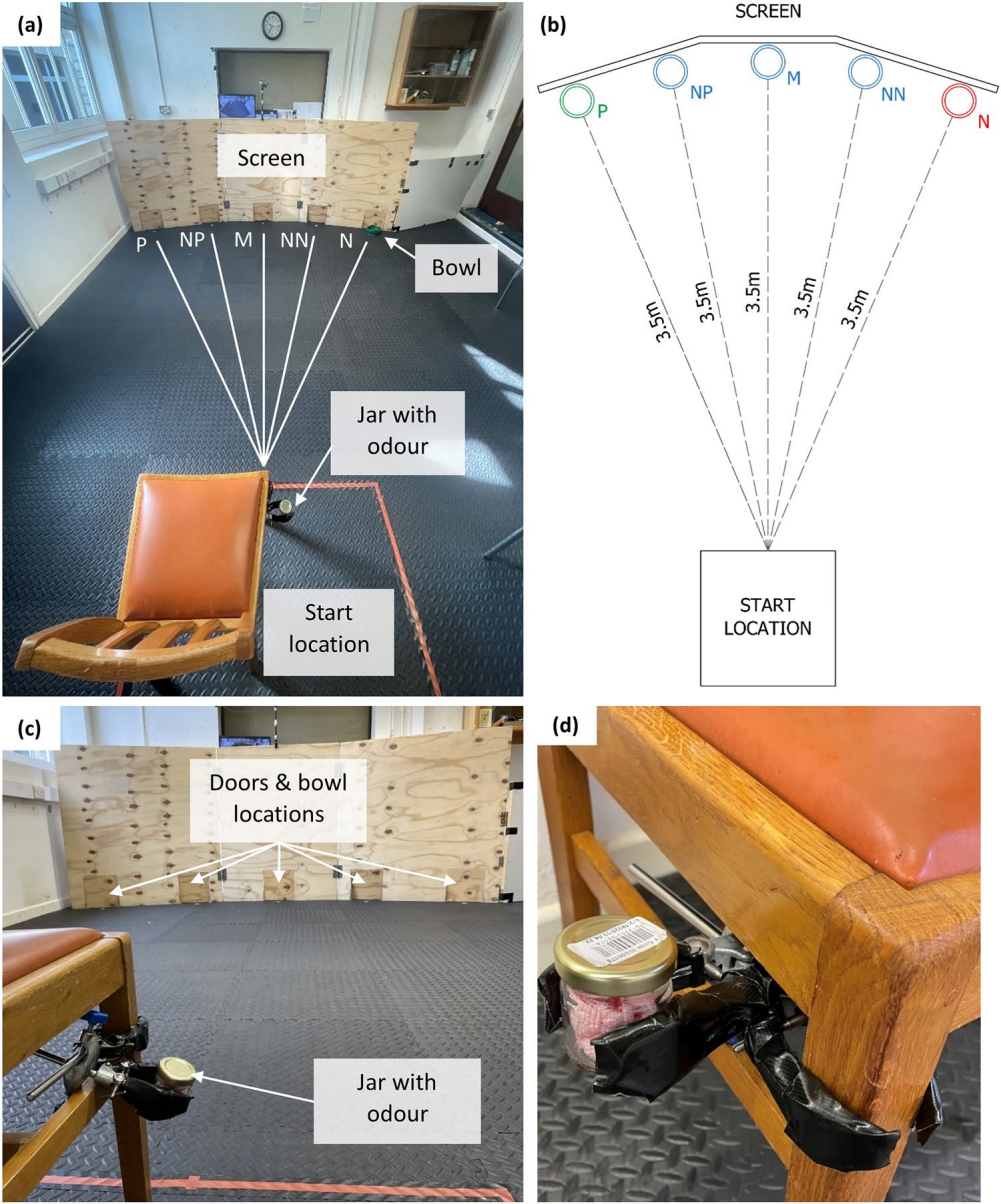
Cognitive bias testing layout. (**a**) Photo of the testing room showing the start location marked by red tape, the jar containing the odour cloth, the screen and five possible bowl locations: P = positive, NP = near-positive, M = middle, NN = near-negative, N = negative. The green bowl used is shown at the N location. (**b**) Scale diagram showing the distance between the start location and bowl locations. (**c**) View of the testing room from the start location, showing the position of the jar, screen, and bowl locations. The bowl was placed either at the P (rewarded) or N (unrewarded) location in the training phase and either at the NP, M, or NN location (unrewarded) in the testing phase. (**d**) Close-up of the jar containing a blank cloth or cloth with stress or relax odour attached using clamps to the chair at the start location.

The cognitive bias protocol used was based on Mendl et al*.* and Hale^57,76^. During the test, owners (acting as handlers) sat at a start location with their dog and faced a screen on the opposite side of the room. The screen developed and provided by Hale^76^ had five doors through which the primary researcher (ZPC) pushed a food bowl whilst remaining behind the screen (Fig. 3a,c). Only one bowl was visible to the dog at any one time (one bowl presentation per trial), and the location of the bowl alternated between the five possible locations (Fig. 3a) over subsequent trials throughout the session. Each possible bowl location was 3.5 m from the start location (Fig. 3b). The screen provided a visual barrier so that the dog was unable to see the researcher preparing the bowl before each trial. Only when the bowl was placed at the positive (P) location was a reward placed in the bowl (reward rate 100%). All other locations never contained a reward (reward rate 0%). When preparing an empty bowl, the researcher “mock” baited the bowl so that any auditory cues of baiting were the same whether the bowl was baited or empty. After placing the bowl through a door, the researcher gave a “release” command, and the handler released their dog from the start location, giving a verbal “go get it” or “ok” prompt. The dog then had the choice to approach or not approach the single bowl (at one of the five locations). The latency to reach the bowl was measured using a manual stopwatch, which was started the moment the handler released their dog and stopped the moment the dog’s head reached the bowl, as observed through a live overhead camera (GoPro Hero 4, GoPro, Inc., San Mateo, CA, USA) connected to a monitor. After the dog ate the reward (if present), a “recall” command was given by the researcher, and the handler called the dog back to the start location. If the dog did not approach the bowl within 30 s, a maximum time of 30 s was recorded, and the “recall” command was given (even if the dog remained at the start location). The handler then reset their dog’s position within the start box in preparation for the next trial. Handlers were able to choose their verbal release prompt but were instructed to keep any verbal prompt and gestures the same for all trials and all sessions. If the dog did not approach the bowl following the prompt, handlers were instructed not to repeat their prompt and to avoid encouraging/insisting that their dog approach, as this could introduce bias and alter the dog’s motivation to approach the bowl. The food rewards were Pedigree Schmackos (turkey flavour) cut into 2 cm pieces. Due to dietary restrictions, one dog received 1 cm cubes of cheese, and another received Pedigree Tasty Minis for puppies instead. All dogs received the same type and volume of food reward in all three sessions. The same bowl was used for all locations so that no distinguishing features of the bowl itself could be used to predict the presence of a reward.

##### Training phase

Each session began with a training phase during which, on each trial, the food bowl was either presented at the positive (P) training location (containing a food reward) or negative (N) training location (empty bowl). The training phase began with two trials at the P location followed by two at the N location, after which the order of P and N trials was pseudorandomised, with a maximum of two consecutive trials at the same location. Training at these two locations was repeated for a minimum of 15 trials and continued until the dog was consistently slower to approach the N location than the P location. This was measured using the following criteria based on the latencies in the preceding six trials (3 × N location and 3 × P location):

1. The *fastest* of three approaches to the N location was ≥ 3 s *slower* than the *slowest* of three approaches to the P location^57^.
2. The *mean* latency of the three approaches to N was > 1 s longer than the *mean* latency of the 3 approaches to P.

A maximum of 50 trials were permitted to meet both these criteria. After passing both criteria, the dog moved on to the testing phase. To shorten the length of subsequent sessions and minimise fatigue, during sessions 2 and 3, the minimum number of training trials was reduced from 15 to six, after which the same criteria were used. The P and N bowl locations were counterbalanced across dogs so that nine dogs were presented with the P location on the right side of the room, and the other nine were presented with the P location on the left side of the room. The location of P and N remained the same for all three sessions for each dog since switching sides in subsequent sessions could introduce ambiguity at these locations and impact the dog’s perception of N and P locations that should be considered “unambiguous”.

##### Testing phase

After successfully passing the training phase, the testing phase began. During the testing phase, dogs were presented with an empty food bowl placed at one of three novel intermediate locations between the P and N positions: near-positive (NP), middle (M), or near-negative (NN) (Fig. 3a,b). These were considered “ambiguous locations” since the dog had no prior rewarded or unrewarded associations with these positions. Bowls presented at these locations never contained a reward (reward rate 0%). During each session, a bowl was presented twice at each ambiguous location. The order of presentation was pseudo-randomised but remained the same for all dogs across all sessions to minimise any effect of order when comparing between sessions. Between each ambiguous bowl presentation, dogs were presented with a full bowl placed at the P location twice and an empty bowl at N twice to reinforce previous associations. Therefore, for each session, there were a total of 26 trials in the testing phase (6 ambiguous and 20 re-enforcement trials).

##### Reward control

To test whether the dog was using olfactory or other cues to determine if a reward was present, at the end of session 3, the dog was presented with a baited bowl at the negative location (N+) and an empty bowl at the positive location (P−). This was only conducted in the final session so that the associations of P and N were consistent until the end of the study.

#### Introducing odour

Before each session, the assigned sample (or blank frozen cloth for session 1) was taken out of the freezer by the primary researcher wearing gloves. The cloth was removed from its bag using forceps and placed in a glass jar, which was sealed immediately with an aluminium screw top. The closed jar was attached to the chair at the start location (Fig. 3d), the position of which was adjusted to each dog’s nose height. The sample was allowed 30 min to reach room temperature before the cognitive bias test began. The jar remained closed for the training phase of each session so that there was no influence of odour on learning the positive and negative cues. Before being given the “release” command, the handler was given the command “jar”, which prompted them to either tap the lid of the jar (training phase) or unscrew and lift the lid off the jar (testing phase). During testing, the lid remained open for 20 s before each trial to allow the odour to be released and detected by the dog. During this time the handler was instructed to keep their dog close to the jar and gently present the jar lid to the dog to sniff, allowing them to inspect and sniff the sample. Handlers were told not to insist the dog sniff the jar or forcefully push the lid towards the dog’s nose, which could be aversive. They were also instructed to remain neutral and not make any gestures or verbal comments towards the jar so as not to create any positive or negative associations with the odour or jar itself. After 20 s, the experimenter repeated the “jar” command and the handler replaced and closed the lid of the jar (or tapped the lid during the training phase). The handler was then prompted to release their dog to start the trial. After recalling their dog from their approach, the opening and closing of the jar (or tapping of the lid) was repeated before releasing them for the next trial. Closing the jar between trials allowed for the accumulation of odour in the headspace of the jar so that the odour was released in repeated bursts at the start of each trial. This gave high odour concentrations at the start of each trial and allowed us to keep the procedure the same for all trials. Opening the jar once at the start of the session would have resulted in a gradual diffusion of the odour potentially rendering it less potent as the session progressed.

Each sample or blank cloth was used only once. After each use, glass jars were put through an enzymatic wash cycle, wiped down with methanol to remove any residual organic odour, and then sterilised in the autoclave before reuse. Before each session, the windows and doors in the testing room were ventilated for 1 h. After each session, the floors were thoroughly cleaned with warm water and an odourless disinfectant cleaner (Safe 4) at 1:100 dilution. An odourless odour eliminator (Dew Products) was then sprayed to remove any residual odours from the samples or dogs. The room was ventilated again for 1 h after cleaning. A minimum of 3 h was allowed between sessions, with a maximum of two sessions per day to minimise odour build-up in the room. There was a minimum of 24 h “wash out” period between sessions with different sample types (stress or relax). If two sessions took place on the same day, the sample donor and odour type were kept the same for both sessions or consisted of a session with a blank cloth followed by a treatment odour.

### Statistical analysis

#### Human stress response

To calculate the change in saliva cortisol for each participant, the pre-test cortisol concentration (stage 3, Fig. 2) was subtracted from the post-test concentration (stage 5) for each condition, giving a post-stress and post-relax cortisol response. To calculate the delayed change in saliva cortisol, the pre-test concentration was subtracted from the post-recovery concentration (stage 6) for each condition, giving a delayed-stress and delayed-relax cortisol response. To calculate the change in mean heart rate, Stress Index and RMSSD for each participant, the baseline value (stage 2) was subtracted from the test period (stage 4) for each condition. To calculate the change in STAI state scores in response to each condition, pre-test scores were subtracted from post-test scores for each participant. Since data for mean heart rate, STAI and Stress Index were normally distributed, paired t-tests were used to compare the change from baseline to the test period/post-test between stress and relax conditions for these three measures. Since data for saliva cortisol and RMSSD were not normally distributed, paired Wilcoxon signed rank tests were used to compare changes from baseline to the test period/post-test between stress and relax conditions for these two measures. T-tests and Wilcoxon tests were run using the stats R core package.

#### Cognitive bias testing

##### Reward control

To test whether the dogs were using olfactory or other cues to determine if a reward was present, we tested the difference between approaching training locations (P and N) when baited or empty. Wilcoxon tests were used to compare the latency to reach the bowl placed at the N location when the bowl was baited (N+) versus empty (N) and the latency to reach the bowl placed at the P location when the bowl was empty (P−) versus baited (P).

##### Likelihood of approaching the bowl

The response to each bowl presentation comprised two components: whether the dog approached the bowl within the time allowed (30 s) and the time taken to reach the bowl (if an approach occurred). To assess these two components combined, Cox proportional hazard models were used. These explored how the fixed effects (described below) affected whether an approach occurred (binary) *and* the time taken to reach the bowl (in seconds). Typically used in survival analysis, a Cox proportional hazards model incorporates both the time to an event (latency to reach bowl) and whether the event occurred or not (approached bowl within 30 s or not) to calculate the likelihood of an event occurring. Combining these, Cox proportional hazards model examines how fixed effects influence the rate at which an event occurs by estimating the hazard ratio (HR) compared to a reference level. In this case, the HR indicates the relative likelihood of the dog approaching the bowl at any given time within 30 s. An HR < 1 indicates a decreased likelihood of approaching the bowl, an HR > 1 indicates an increased likelihood of approaching the bowl, and an HR = 1 indicates no effect compared to the reference level. To assess whether dogs successfully learned to discriminate between the five bowl locations (P, NP, M, NN, N) a Cox proportional hazards model was run to determine the effect of bowl location on the likelihood of the dog approaching, with the P location used as a reference level. To assess the effect of other variables within each location, the dataset was filtered by location, and Cox proportional hazards models were run at each location for the following fixed effects: treatment odour (blank cloth, stress, relax), session number (session 1, session 2, session 3), whether the dog was a registered teaching dog (yes/no), attachment type (person present, another person, multiple people, no attachment), dog emotional sensitivity (1–5), handler relaxation score (1–10) and sample donor. Dog ID was included as a random effect to control for individual differences in running speed (i.e. Cox mixed-effects analysis). To reduce variables within the model and select the model with the best fit, stepwise deletion of terms based on Akaike’s Information Criterion (AIC) was used using the drop1 function in R. Tukey’s post-hoc pairwise comparisons based on estimated marginal means (least-squares means) were run on significant terms. To assess the effect of odour within each order of presentation, a stratification term of treatment order (order 1: stress → relax and order 2: relax → stress) was included. Information on the number of trials in which no approach was made within 30 s to each location can be found in Supplementary Table S2. Analyses were run using the coxme (ver. 2.2-18.1, the LGPL-2 licence, https://cran.r-project.org/src/contrib/Archive/coxme/)^77^, survival (ver. 3.3-1, the LGPL (≥ 2) licence, https://cran.r-project.org/src/contrib/Archive/survival/)^78^ and emmeans (ver. 1.7.4-1, the GPL-2 | GPL-3 licence, https://cran.r-project.org/src/contrib/Archive/emmeans/)^79^ R packages. Plots were constructed using the ggplot2 (ver. 3.4.4, the MIT licence, https://cran.r-project.org/src/contrib/Archive/ggplot2/)^80^, ggstatsplot (ver. 0.11.1, the GPL-3 licence, https://cran.r-project.org/src/contrib/Archive/ggstatsplot/)^81^ and ggpubr (ver. 0.4.0, the GPL-2 licence, https://cran.r-project.org/src/contrib/Archive/ggpubr/)^82^ R packages. All analyses and plots were produced using R Studio version 2023.06.2+561.

## Results

### Stress induction in human odour donors

Six donors showed an increase in salivary cortisol from baseline to immediately post-stress test. Of these, two donors showed a subsequent decrease in salivary cortisol post-recovery (20-min post-test), which was lower than their baseline. Therefore, only four participants showed a sustained increase in salivary cortisol from baseline to both post-test and post-recovery time points. All eleven donors showed a decrease in salivary cortisol from baseline to post-relax test, and ten showed a decrease from baseline to post-relax recovery. Paired Wilcoxon signed rank tests found that the change in salivary cortisol concentration from baseline to immediately post-test was not statistically different between conditions (V = 53, p = 0.083). However, the change from baseline to post-recovery was significantly different between stress (mean change =  + 0.088 ug/dL, SD = 0.236) and relax (mean change =  − 0.078 ug/dL, SD = 0.057) conditions (V = 64, p = 0.003). All donors showed an increase in mean heart rate from baseline during the stress condition and a decrease from baseline during the relax condition. There was a significant difference in the change in mean heart rate between stress (mean change =  + 21.6 bpm, SD = 7.6) and relax (mean change = − 4.9 bpm, SD = 2.8) conditions (t(10) = 14.71, p < 0.001). All donors showed an increase in Stress Index during the stress condition, and seven showed a decrease during the relax condition. There was a significant difference in the change in Stress Index between stress (mean change =  + 4.6, SD = 2.7) and relax (mean change = – 1.9, SD = 2.6) conditions (t(10) = 9.03, p < 0.001). All participants showed a decrease in RMSSD during the stress test, and eight showed an increase during the relax condition. There was a significant difference in the change in RMSSD between stress (mean change = –27.7 ms, SD = 20.2) and relax (mean change =  + 25.9 ms, SD = 46.8) conditions (V = 0, p < 0.001). All donors showed an increase in STAI state anxiety scores from pre- to post-stress test and ten showed a decrease from pre- to post-relaxing activity. There was a significant difference in the change in STAI state scores between stress (mean change =  + 22.4, SD = 8.0) and relax (mean change = – 3.6, SD = 3.7) conditions (t(10) = 7.97, p < 0.001). See Supplementary Fig. S1 for plots of these results.

#### Selecting odour samples

When selecting odour samples for cognitive bias testing, we selected donors who consistently showed a stress response (compared to their own baseline) across all measures. Since all eleven donors showed changes consistent with stress response for heart rate, Stress Index, RMSSD and STAI scores in response to the stress test, we primarily based our selection on the salivary cortisol response. We first selected the four donors who showed a sustained increase in salivary cortisol from baseline to post-stress test and post-recovery. Of these, one donor was excluded since they showed a mild increase in cortisol in response to the relaxing condition. We, therefore, selected the remaining three participants (two women and one man).

### Cognitive bias testing

#### Reward control

There was no significant difference in the latency to approach the P location dependent on whether the bowl was baited or empty (W = 1613.5, p = 0.979). There was a significant difference in the latency to approach the N location when the bowl was empty compared to baited (W = 1112.5, p = 0.015). However, the mean (± SEM) time to approach the baited bowl (N+) was longer (26.5 s ±1.9) than the empty bowl (N) (19.6 s ±0.9), indicating the presence of the reward at this location did not cause the dogs to approach faster. These results confirm that dogs were not using olfactory cues to determine if a bowl contained food and relied on the learned association between location and the presence/absence of a reward.

#### Likelihood of approaching the bowl

The Cox proportional hazards model that provided the best fit (based on AIC) included interactions between bowl location and odour treatment, between bowl location and session number and between bowl location and whether the dog was a registered teaching dog (yes/no). However, we found no significant differences between teaching and non-teaching dogs within any odour treatment (see Supplementary Analysis in Supplementary Information). There was no significant effect of dog emotional sensitivity, attachment type, handler relaxation scores or sample donor on the likelihood of dogs approaching the bowl at any location. These variables were dropped from the model during stepwise deletion of terms.

#### Discrimination between bowl locations

**Figure 4:**
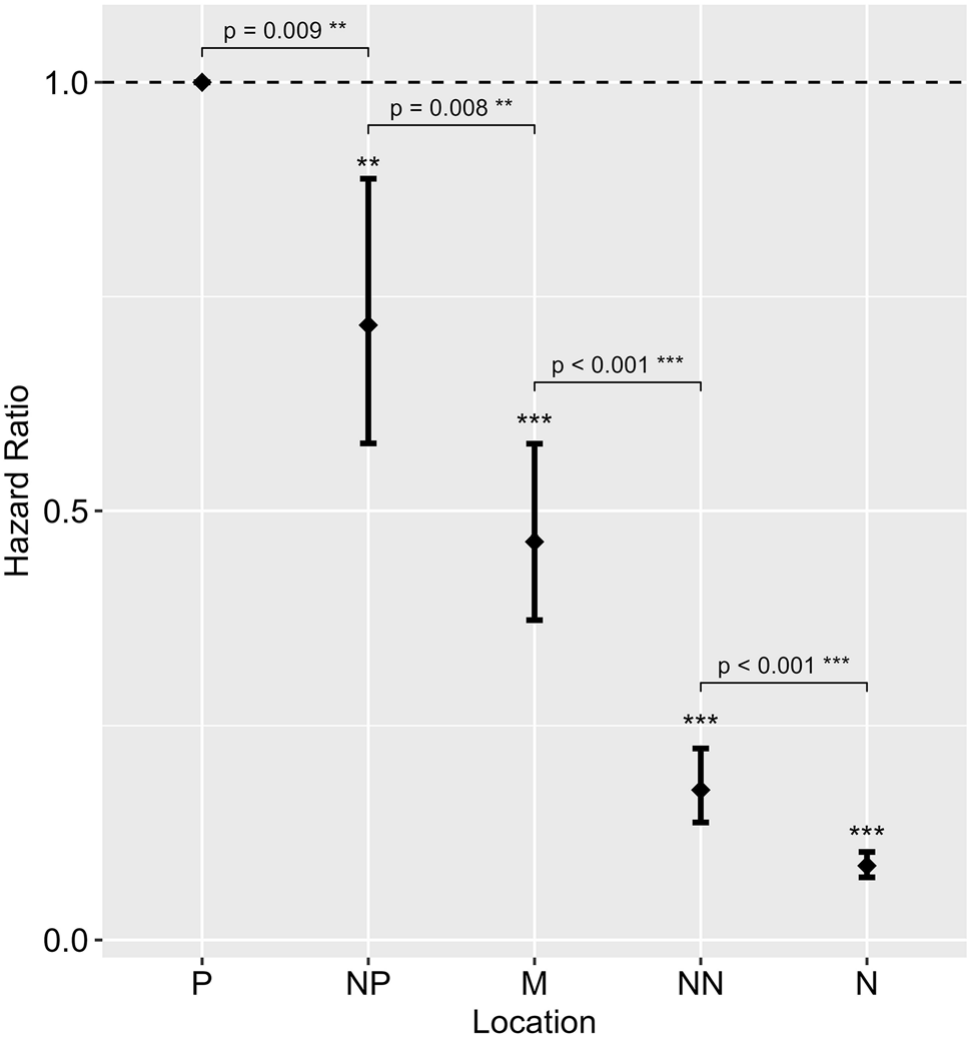
Plot showing the results of the Cox mixed-effects analysis for the effect of location on the likelihood of approaching the bowl. The horizontal dashed line indicates the reference level (Hazard Ratio 1): positive location (P), to which the likelihood of approaching the bowl when placed at the near-positive (NP), middle (M), near-negative (NN), and negative (N) locations were compared. Results show a decrease in likelihood of approaching the bowl at each location (Hazard Ratio < 1) compared to the P location. Significance levels are indicated by the asterisks above each bar. Error bars represent 95% confidence intervals. Pairwise differences between locations are indicated by the square brackets. Significance codes: ***p < 0.001, **p < 0.01, *p < 0.05.

Compared to the P location (reference level), Cox mixed-effects analysis showed a significant decrease in the likelihood of approaching the bowl at NP (HR = 0.72, p = 0.003), M (HR = 0.47, p < 0.001), NN (HR = 0.18, p < 0.001) and N (HR = 0.09, p < 0.001) (Fig. 4). Post-hoc pairwise comparisons showed significant differences between all bowl locations (p < 0.01), indicating dogs were successful at discriminating between locations as required by the test paradigm.

#### Does the odour of human stress or relaxation affect the likelihood of approaching the three ambiguous bowl locations?

**Figure 5:**
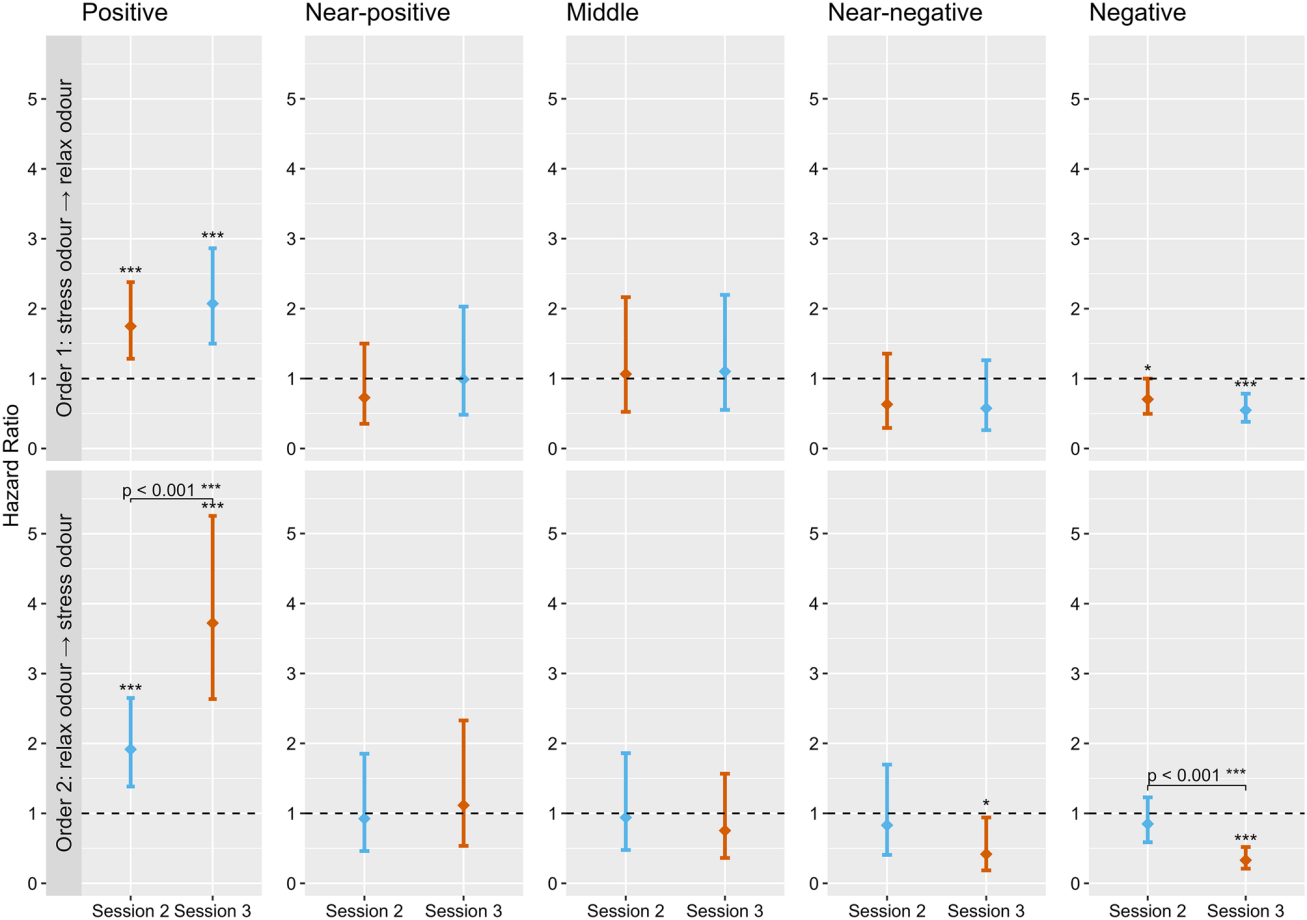
Plots showing the results of the Cox mixed-effects analysis for the effect of session on the likelihood of approaching the bowl (Hazard Ratio) when placed at the positive, near-positive, middle, near-negative and negative locations. The top five plots show results for dogs exposed to the stress odour (orange) before the relax odour (blue) (order 1). The bottom five plots show results for dogs exposed to the relax odour before the stress odour (order 2). The horizontal dashed lines indicate the reference level (Hazard Ratio = 1) that represent the blank cloth odour session (session 1), to which the likelihood of approaching during sessions 2 and 3 are compared. Error bars represent 95% confidence intervals. Significant differences in the likelihood of approaching during session 2 or 3 compared to blank cloth odour are indicated by the asterisks above individual bars. Pairwise differences between sessions 2 and 3 are indicated by the square brackets. Significance codes: ***p < 0.001, **p < 0.01, *p < 0.05.

Cox mixed-effects analysis showed that of the three ambiguous bowl locations (NP, M and NN), there was a significant main effect of stress odour only on the likelihood of approaching the bowl when placed at the NN location (HR = 0.53, p = 0.027) with dogs exposed to stress odour being less likely to approach than those exposed to blank cloth odour (Supplementary Fig. S2). There was also a significant main effect of session number with dogs being less likely to approach the NN location in session 3 (HR = 0.48, p = 0.013) compared to session 1 (Supplementary Fig. S3). On closer examination, we found differences in these effects depending on the treatment order. When looking at the interaction between odour and session, Cox mixed-effects analysis (stratified by treatment order) showed that only dogs exposed to the stress odour in session 3 (order 2) were significantly less likely to approach the NN location in that session (HR = 0.42, p = 0.035) compared to when exposed to blank cloth odour (Fig. 5). Whereas dogs exposed to stress odour in session 2 (order 1), showed no significant effect of either odour or session number on the likelihood of approaching the bowl when placed at the NN location (Fig. 5). Post-hoc pairwise comparisons found no significant difference between stress and relax odours in either treatment order at the NN location, and there were no significant effects of odour or session at NP or M locations.

#### Does the odour of human stress or relaxation affect the likelihood of approaching the two training locations?

For training locations (P and N), Cox mixed-effects analysis found that compared to the blank cloth odour, both odour and session had significant main effects on the likelihood of the dog approaching both P and N locations (Supplementary Figs. S2, S3). Again, we found differences in these effects depending on the treatment order. When looking at the interaction between odour and session, Cox mixed-effects analysis (stratified by treatment order) found that compared to the blank cloth odour, dogs exposed to the stress odour before the relax odour (order 1) were significantly more likely to approach the bowl when placed at the P location in both session 2 (HR = 1.75, p < 0.001) and session 3 (HR = 2.07, p < 0.001) (Fig. 5). Similarly, dogs presented with the relax odour before the stress odour (order 2) were significantly more likely to approach the bowl when placed at the P location in session 2 (HR = 1.89, p < 0.001) and session 3 (HR = 3.66, p < 0.001). However, post-hoc pairwise comparisons found that the difference between sessions 2 and 3 was only significant for dogs presented with the relax odour before the stress odour (order 2) (p < 0.001), with the likelihood of approaching the P location increasing significantly in session 3 in the presence of stress odour (Fig. 5). For the N location, Cox mixed-effects analysis found that compared to the blank cloth odour, dogs presented with the stress odour before the relax odour (order 1) were significantly less likely to approach the bowl when placed at the N location in both session 2 (HR = 0.70, p = 0.050) and session 3 (HR = 0.55, p = 0.001) (Fig. 5). In contrast, dogs presented with the relax odour before the stress odour (order 2) were only significantly less likely to approach the bowl when placed at the N location in session 3, when the stress odour was present (HR = 0.33, p < 0.001) (Fig. 5). Post-hoc pairwise comparisons found that the likelihood of approaching the N location was only significantly different between sessions 2 and 3 when dogs were exposed to the relax odour before stress odour (order 2) (p < 0.001), with the likelihood of approaching the N location decreasing significantly in session 3 in the presence of stress odour (Fig. 5).

## Discussion

In this study, we examined whether human stress and relax odours affected dogs’ likelihood of approaching three ambiguously located food bowls in a cognitive bias paradigm. In addition, we examined the effect of these odours on dogs’ likelihood of approaching known positively and negatively associated training bowl locations. Sweat and breath samples for stress and relax odours were collected from three unfamiliar volunteers during a stress test and relaxing activity, confirmed by changes in mean heart rate, HRV, STAI scores and salivary cortisol. Our findings indicate that compared to blank cloth odour, the presence of human stress odour in session 3 caused dogs to exhibit risk-reduction behaviours and a more ‘pessimistic’ response to the possibility of a reward at the ambiguous NN location. This suggests that dogs in this group were in a more negative affective state in the presence of stress odour than blank cloth odour.

Results showed significant main effects of both odour and session number at one of the three ambiguous bowl locations. Compared to the presence of blank cloth odour in session 1, dogs were approximately half as likely to approach the bowl when placed at the ambiguous NN location when the stress odour was present (HR = 0.53) (Supplementary Fig. S2) and during session 3 (HR = 0.48) (Supplementary Fig. S3). On closer examination, this decrease in the likelihood of approaching the bowl at the NN location was only significant when the stress odour was present in session 3 (HR = 0.42), indicating an interaction between odour and session. We also observed significant main effects of odour and session at the two training locations (P and N) (Supplementary Figs. S2, S3). Here, we found that compared to the blank cloth odour in session 1, dogs were significantly more likely to approach the bowl when placed at the P location and less likely to approach when placed at the N location in the presence of both odours and both subsequent sessions. Again, the results of the Cox mixed-effects analysis showed significant differences in the likelihood of approaching the bowl at these two training locations, depending on the odour treatment order (Fig. 5). Pairwise comparisons showed that only dogs exposed to the stress odour in session 3 showed a significant increase in the likelihood of approaching the P location and a significant decrease in the likelihood of approaching the N location between sessions 2 and 3. Furthermore, results for the N location show that while both odours significantly decreased the likelihood of approaching in session 3 compared to session 1, only the stress odour significantly decreased the likelihood of approaching in session 2 compared to session 1. These results indicate that the stress odour in session 3 had the greatest effect at all three locations (NN, N and P).

The interaction between odour and session suggests that both exposure to the stress odour and a learning effect caused by repeated testing influenced dogs’ responses. The latter effect has previously been shown to occur in sheep^83^ and in dogs^75^, where Wilson et al. found that repeated cognitive bias testing (up to five sessions) resulted in an increase in latency to approach ambiguous locations and a decrease in the number of approaches. This is likely due to animals learning that ambiguous locations are never rewarded, so they begin to treat them as negative cues. To ameliorate this effect, in the current study, we reduced the number of presentations at each ambiguous location from three (as originally described by Mendl et al.^57^ and used by Wilson et al.^75^) to two per session. This provided a balance between reducing the learning effect and minimising the risk of spurious results from a single presentation. Although we observed a significant decrease in the likelihood of approaching NN between sessions 1 and 3 in dogs exposed to stress odour in session 3 (order 2), we did not see a decrease in the likelihood of approaching between sessions 1 and 2 or between sessions 2 and 3. We also did not see a significant effect of session number in dogs exposed to stress odour in session 2 and relax odour in session 3 (order 1). Unlike Wilson et al. we did not observe any effect of learning over sessions at the NP or M locations, suggesting our modification may have been effective at minimising learning effects at these two ambiguous locations. Although the effects at N and P were not reported by Wilson et al., the significant decrease in the likelihood of approaching N and the significant increase in the likelihood of approaching P between sessions in the current study is likely also due to a learning effect. This suggests that dogs continued learning about these reinforced locations during testing. The inclusion of a third “control” group of dogs only exposed to blank cloth odour over repeated sessions should be considered in future studies and would allow for a better interpretation of the learning effects in repeated cognitive bias testing. Nevertheless, the differences observed between the two treatment orders used in this study suggest the results are not due to learning effects alone.

The change in response from the blank cloth odour in session 1 was greatest when the stress odour was present in session 3 for NN, N and P locations, suggesting an amplifying or additive effect of session 3 and the stress odour. As described in a review by Thomas et al., acute stress has differing effects on memory formation depending on the timing and magnitude of the stressor^84^. Stress during task acquisition has been shown to improve memory encoding and learning^85^. This is thought to be due to the effect that acute stress has on narrowing focus to more “important” details and ignoring irrelevant ones^86^. Considering the opposite effects observed at P and N locations, which were greatest in the presence of the stress odour in session 3, it’s possible that the stress odour improved dogs’ ability to learn the distinction between positive and negative cues faster within the session. This is supported by the findings of Sumegi et al., who found that inducing stress in either the owner or the dog significantly improved dogs’ performance in a working memory task compared to dogs where neither dog nor owner was stressed^87^. Zubedat et al. found that detection dogs were quicker to detect an explosive when their handler had been stressed (told they were being reassigned to another unit or investigated by military police) compared to unstressed^88^, suggesting mild handler stress may influence working performance in dogs. However, it is important to remember that very high or chronic stress levels can lead to learning and memory impairment^89–92^.

Another possible explanation for the interaction between odour and session is a carry-over or order effect. We found that the differences between sessions 2 and 3 at N and P training locations were only significant when the stress odour was introduced *after* the relax odour (order 2), but not when stress odour was introduced *before* relax odour (order 1). In addition, the significant effect of session on the likelihood of approaching the NN location was only observed when the stress odour was presented after the relax odour. The human literature suggests that while stress during learning improves subsequent memory recall, stress during memory recall (but not learning) may impair memory recall^93,94^. Therefore if, as in humans, stress enhanced dogs’ ability to learn which locations contained rewards but reduced their ability to recall locations from previous sessions, we would expect dogs exposed to stress odour in session 2 to have improved memory recall in session 3 (when stress odour was not present) and dogs exposed to stress odour during memory recall in session 3 to have reduced memory from the previous session. However, this was not observed. This could suggest that dogs exposed to stress odour first did most of their learning about the N and P locations in session 2 and maintained that into session 3, increasing slightly but not significantly, i.e. they reached their “maximum learning” by session 2. Meanwhile, dogs with reverse order showed a significant change in the likelihood of approaching N and P between sessions 2 (relax odour) and 3 (stress odour), suggesting continued learning across these sessions. Alternatively, the stress odour may have influenced learning *within* a session but not memory recall in the following session.

Since the decrease in likelihood of approaching NN was similar to the results for the N location, it’s possible dogs made a spatial generalisation between NN and N locations, where NN was considered unlikely to contain a reward due to its proximity to N. Taken together with the increase in likelihood of approaching the P location with both odours, these opposite effects could be explained by a change in how dogs evaluated risk and valued rewards. Negative emotional states have been shown to cause risk aversion in humans, causing individuals to be less willing to risk disappointment if they perceive a reward to be unlikely^47–49^. This risk-reduction behaviour is thought to be a way to conserve energy and avoid disappointment^95–98^. Mildly negative states have also been shown to increase reward valuation, causing individuals to value rewards more when they are considered likely^95–98^. This is thought to be a way of “relieving” some of the negative emotions by obtaining a positive reward^95–98^ and is in contrast to severe or chronic negative states that result in a devaluation of rewards (anhedonia)^52,99–101^. If the presence of the stress odour caused dogs to experience a mild negative emotional state, they may have been less willing to “risk” approaching a location where they consider a reward to be *unlikely* (N or NN), i.e., risk-reduction behaviour. However, when presented with a bowl at a location they consider a reward to be *likely* (P), they may have shown increased anticipation and desire for that reward and, therefore, approached the bowl faster, i.e., increased risk-taking behaviour.

Since the odours used in this study were from unfamiliar people, our findings suggest dogs may detect and respond to an olfactory “stress signal” shared across humans^43–45,102^ rather than responding to a familiar odour with a learned response. This is supported by the findings of D’Aniello et al., who found that both puppies (between 3 and 6 months) and adult dogs respond to the odour of human fear by showing an increase in owner-directed behaviours, whereas responses to the odour of human “happiness” were only observed in adult dogs^103^. This suggests that whereas the interpretation of odours associated with positive human emotions, such as happiness, may require learning, the interpretation of odours associated with negative human emotions, such as fear, maybe more innate.

## Conclusion

This is the first study to test how the odour of human stress and relaxation affects an indicator of emotional state in dogs. Using a cognitive bias paradigm, we found that human stress odour significantly decreased dogs’ likelihood of approaching an ambiguous cue at a near-negative location, but only in the third test session. An effect that was not observed with the relaxed odour. Dogs were significantly less likely to approach the bowl at the negative training location and more likely to approach the bowl at the positive training location with repeated testing, suggesting a learning effect. However, differences at training locations between all three sessions were only significant when the stress odour was presented in session three, suggesting an interaction effect between odour and learning. These findings may indicate an effect of human odour on the emotional state of dogs and/or how dogs perceive risks and rewards. If the odour from stressed humans affects a dog’s emotional state, perception of rewards, or ability to learn, it suggests that stress may not just travel down the lead (as is often stated) but also through the air. These findings highlight the need to consider dogs’ training and working environments from an olfactory perspective.

### Supplementary Information

Supplementary Information.

## Data Availability

The anonymised data collected are available as open data via the University of Bristol online data repository: https://data.bris.ac.uk/ (10.5523/bris.25y89fousbjcy21d9cjcgnqjg9).
