# Supplementary material for: The odour of an unfamiliar stressed or relaxed person affects dogs’ responses to a cognitive bias test
Source: Sci Rep. 2024 Jul 22;14:15843. doi: 10.1038/s41598-024-66147-1 (PMC11263577; doi:10.1038/s41598-024-66147-1)
Supplement: Supplementary file 1 — Supplementary Information. [file 41598_2024_66147_MOESM1_ESM.docx]

The odour of an unfamiliar stressed or relaxed person affects dogs’ responses to a cognitive bias test

Z Parr-Cortes, CT Müller, L Talas, M Mendl, C Guest & NJ Rooney

Supplementary Information

**Supplementary Table S1.** Table showing sample allocation for dogs in phase 2.

| **Group** | **Sample donor** | **Order** | **Odour type** | | | **Number of dogs (n)** |
| --- | --- | --- | --- | --- | --- | --- |
|  |  |  | **Session 1** | **Session 2** | **Session 3** |  |
| Group 1 | Participant 1 | Order 1 | Blank | Stress | Relax | 3 |
|  |  | Order 2 | Blank | Relax | Stress | 3 |
| Group 2 | Participant 2 | Order 1 | Blank | Stress | Relax | 3 |
|  |  | Order 2 | Blank | Relax | Stress | 3 |
| Group 3 | Participant 3 | Order 1 | Blank | Stress | Relax | 3 |
|  |  | Order 2 | Blank | Relax | Stress | 3 |
|  |  |  |  |  | Total | 18 |

**Supplementary Table S2.** Table showing the number of trials during the testing phase in which the dog did not approach the bowl within 30 s. During each session, dogs had 10 trials at each training location: positive (P) and negative (N) and 2 trials at each ambiguous location: near-positive (NP), middle (M) and near-negative (NN). The total number of dogs in each treatment order group was 9. Therefore, the total number of trials for each session in each treatment order group was n = 90 for P and N locations and n = 18 for NP, M and NN locations.

| **Treatment order** | **Session number** | **Odour** | **Number of trials with no approach to the bowl at each location** | | | | |
| --- | --- | --- | --- | --- | --- | --- | --- |
|  |  |  | **P**  **(n = 90)** | **NP**  **(n = 18)** | **M**  **(n = 18)** | **NN**  **(n = 18)** | **N**  **(n = 90)** |
| Order 1  Stress -> Relax  (9 dogs) | Session 1 | Blank | 2 | 1 | 1 | 1 | 19 |
|  | Session 2 | Stress | 2 | 0 | 2 | 4 | 33 |
|  | Session 3 | Relax | 1 | 1 | 1 | 6 | 37 |
| Order 2  Relax -> Stress  (9 dogs) | Session 1 | Blank | 3 | 0 | 0 | 2 | 28 |
|  | Session 2 | Relax | 4 | 1 | 1 | 3 | 36 |
|  | Session 3 | Stress | 0 | 1 | 2 | 8 | 62 |

**Supplementary Figure S1**


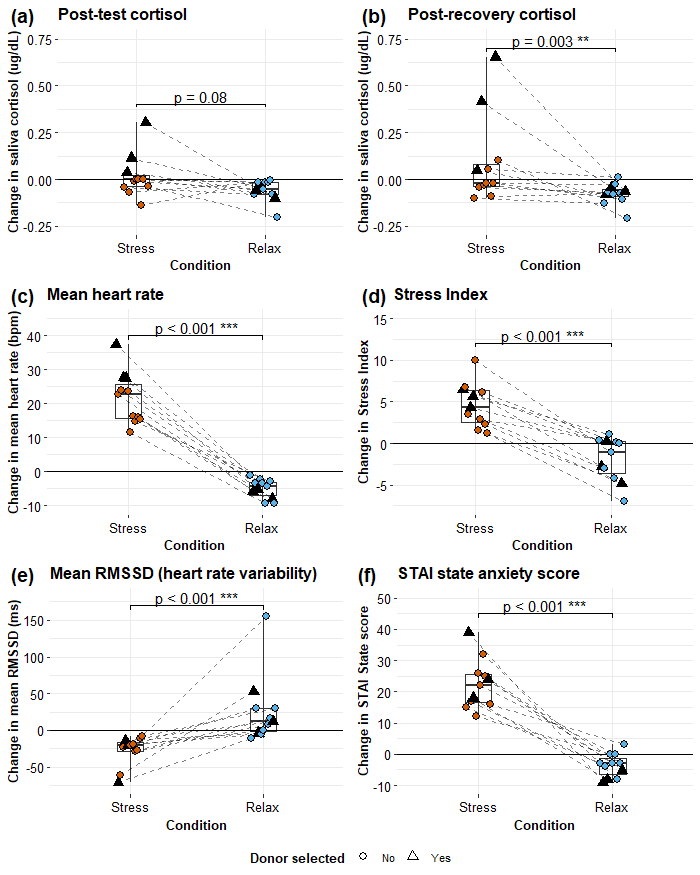


**Supplementary Figure S1.** Boxplots showing differences between stress (orange) and relax (blue) conditions for changes in donors’ **(a)** salivary cortisol from baseline to immediately after the test period, **(b)** salivary cortisol from baseline to post-recovery (20 min after the test period), **(c)** mean heart rate from baseline to the test period, **(d)** Stress Index from baseline to the test period, **(e)** mean RMSSD (root mean square of the successive differences) from baseline to the test period and **(f)** STAI state anxiety score from before to after the test period. Donors whose samples were selected for cognitive bias testing are shown as black triangles. Boxes represent interquartile ranges and medians (a, b and e) or means (c, d and f). The dashed lines represent paired samples for each donor. The solid horizontal lines at 0 represent the baseline calculated for each donor and each condition. Significance codes: *** p < 0.001, ** p < 0.01, * p < 0.05.


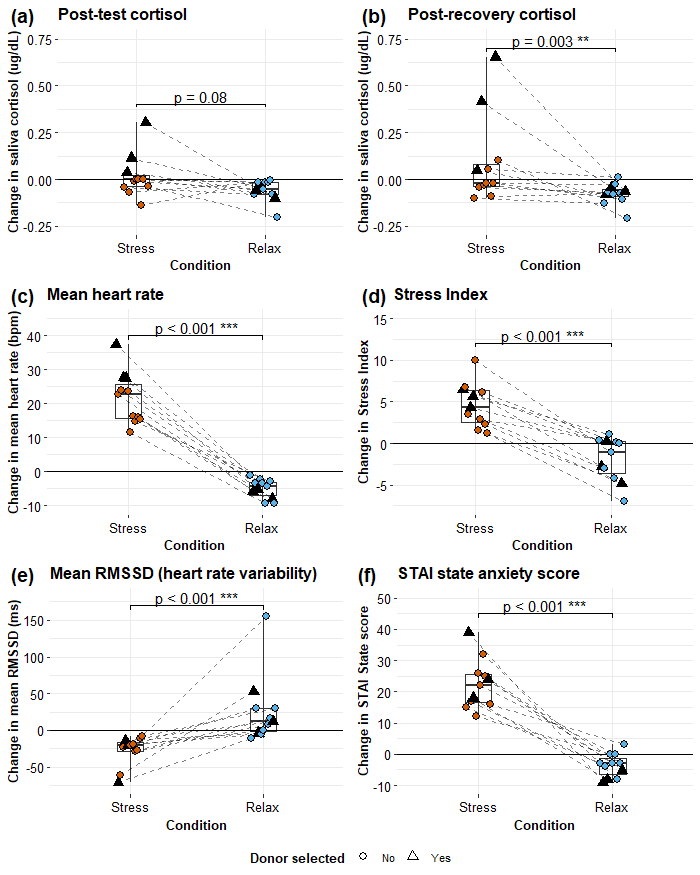


**Supplementary Figure S2**


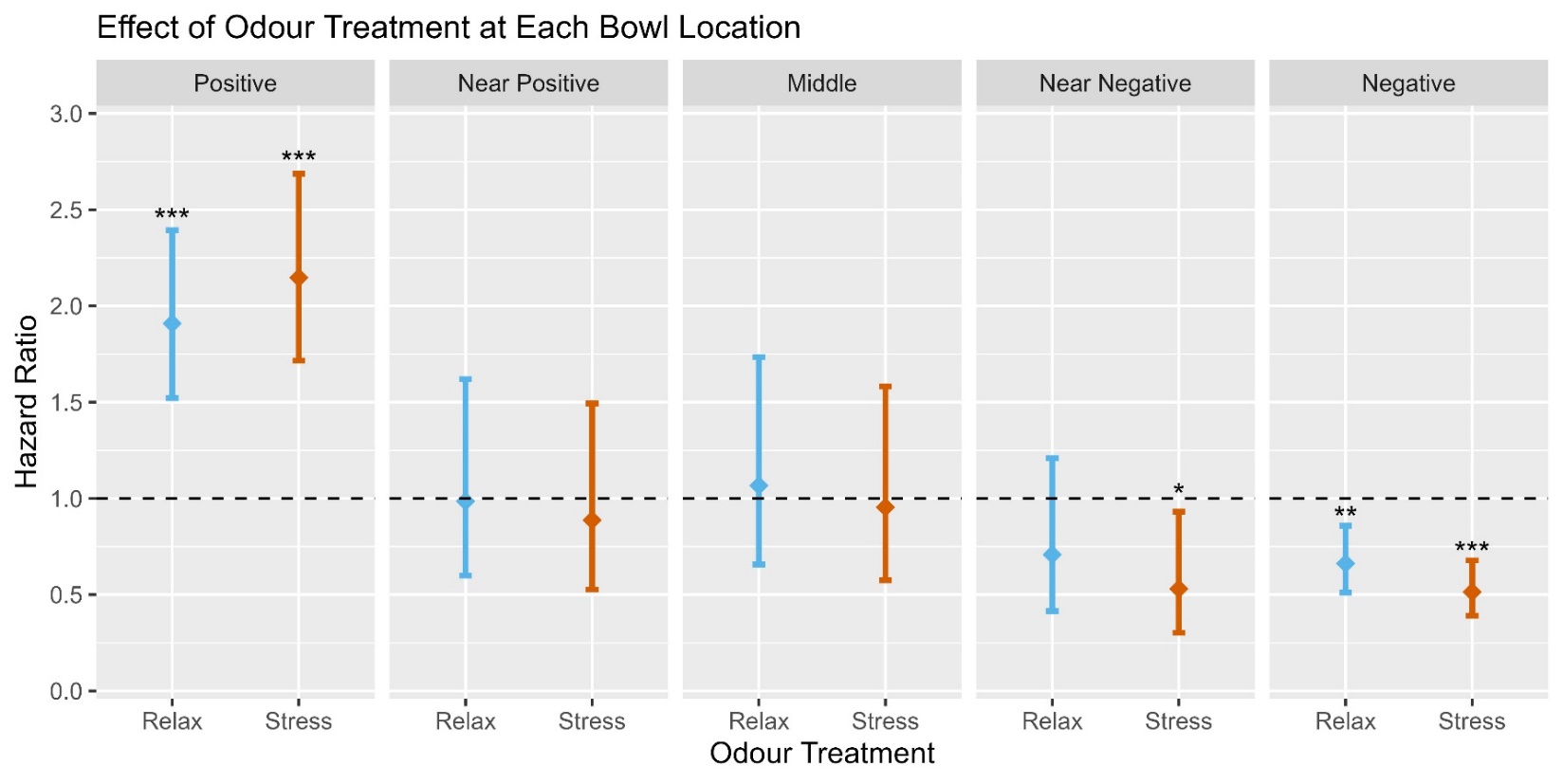


**Supplementary Figure S2.** Plots showing the results of the Cox mixed-effects analysis for the effect of odour on the likelihood of approaching the bowl at each location. The dashed line indicates the reference level (Hazard Ratio = 1): blank cloth odour, to which the likelihood of approaching during exposure to relax odour (blue) and stress odour (orange) are compared. Significant differences in the likelihood of approaching during stress or relax odour exposure compared to baseline are indicated by the asterisks above individual bars. Error bars represent 95% confidence intervals. Significance codes: *** p < 0.001, ** p < 0.01, * p < 0.05.


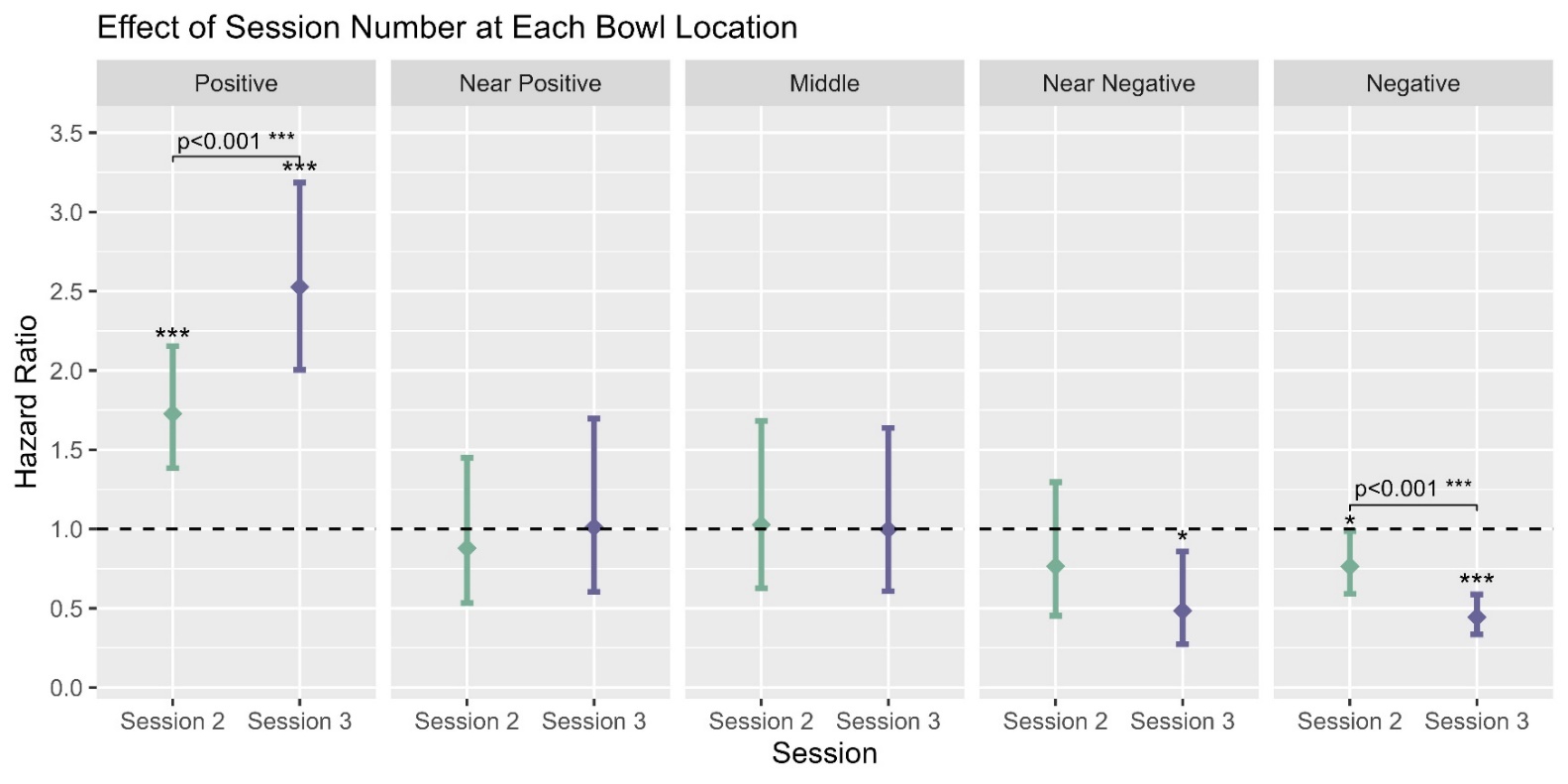


**Supplementary Figure S3.** Plots showing the results of the Cox mixed-effects analysis for the effect of session on the likelihood of approaching the bowl at each location. The dashed line indicates the reference level (Hazard Ratio = 1): session 1, to which the likelihood of approaching during session 2 (green) and session 3 (purple) are compared. Significant differences in the likelihood of approaching during session 2 or 3 compared to baseline are indicated by the asterisks above individual bars. Error bars represent 95% confidence intervals. Pairwise differences between sessions are indicated by the square brackets. Significance codes: *** p < 0.001, ** p < 0.01, * p < 0.05.

**Supplementary Figure S3**

## **Supplementary Analysis -** Teaching versus non-teaching dogs

Overall, teaching dogs were found to be significantly less likely than non-teaching dogs to approach a bowl placed at locations NP (HR = 0.2, p = 0.008) and NN (HR = 0.44, p = 0.025) (Supplementary Fig. S4). However, pairwise comparisons found no significant differences between teaching and non-teaching dogs within any odour treatment (Supplementary Fig. S5), indicating no differences in the responses of teaching and non-teaching dogs to odour treatment.


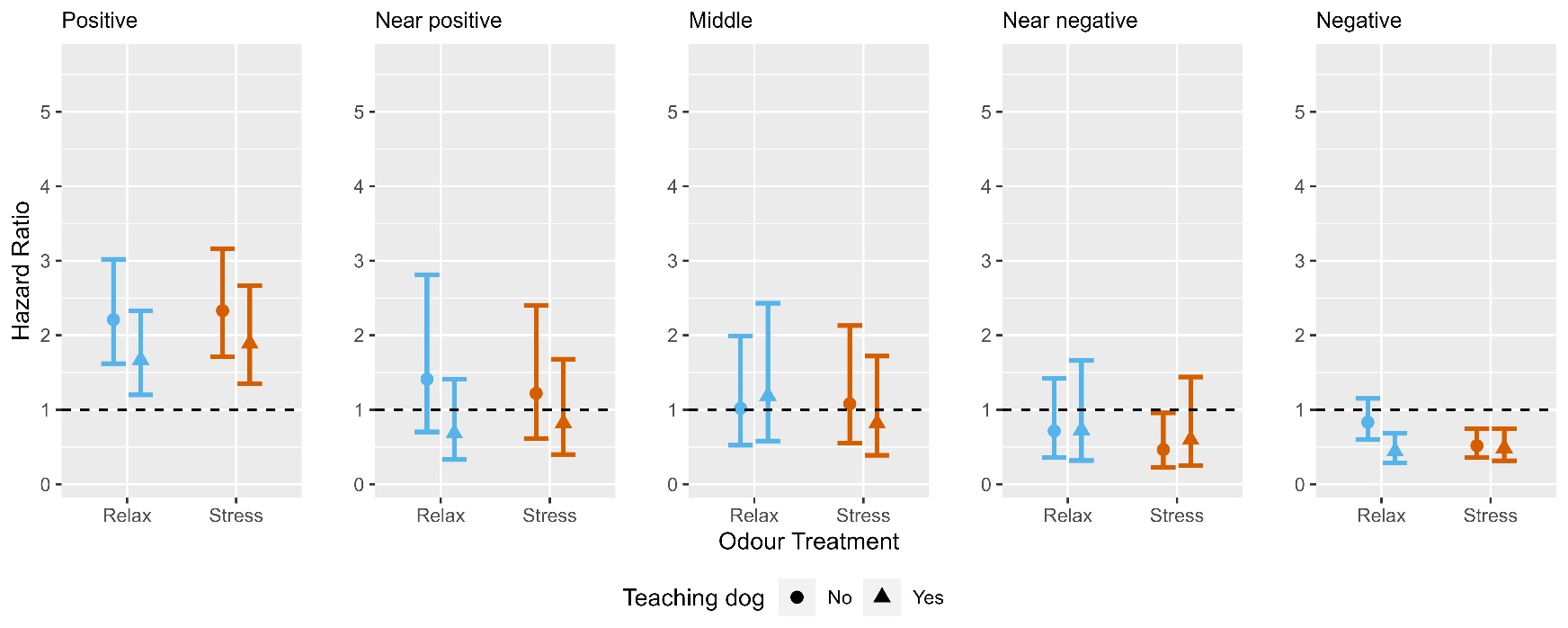


**Supplementary Figure S5.** Plots showing the results of the Cox mixed-effects analysis for the effect of odour treatment: relax (blue) and stress (orange) on the likelihood of approaching the bowl at each location for non-teaching dogs (circles) and teaching dogs (triangles). The dashed line indicates the reference level (Hazard Ratio = 1): blank cloth odour, to which the likelihood of approaching during each odour treatment was compared. Error bars represent 95% confidence intervals.


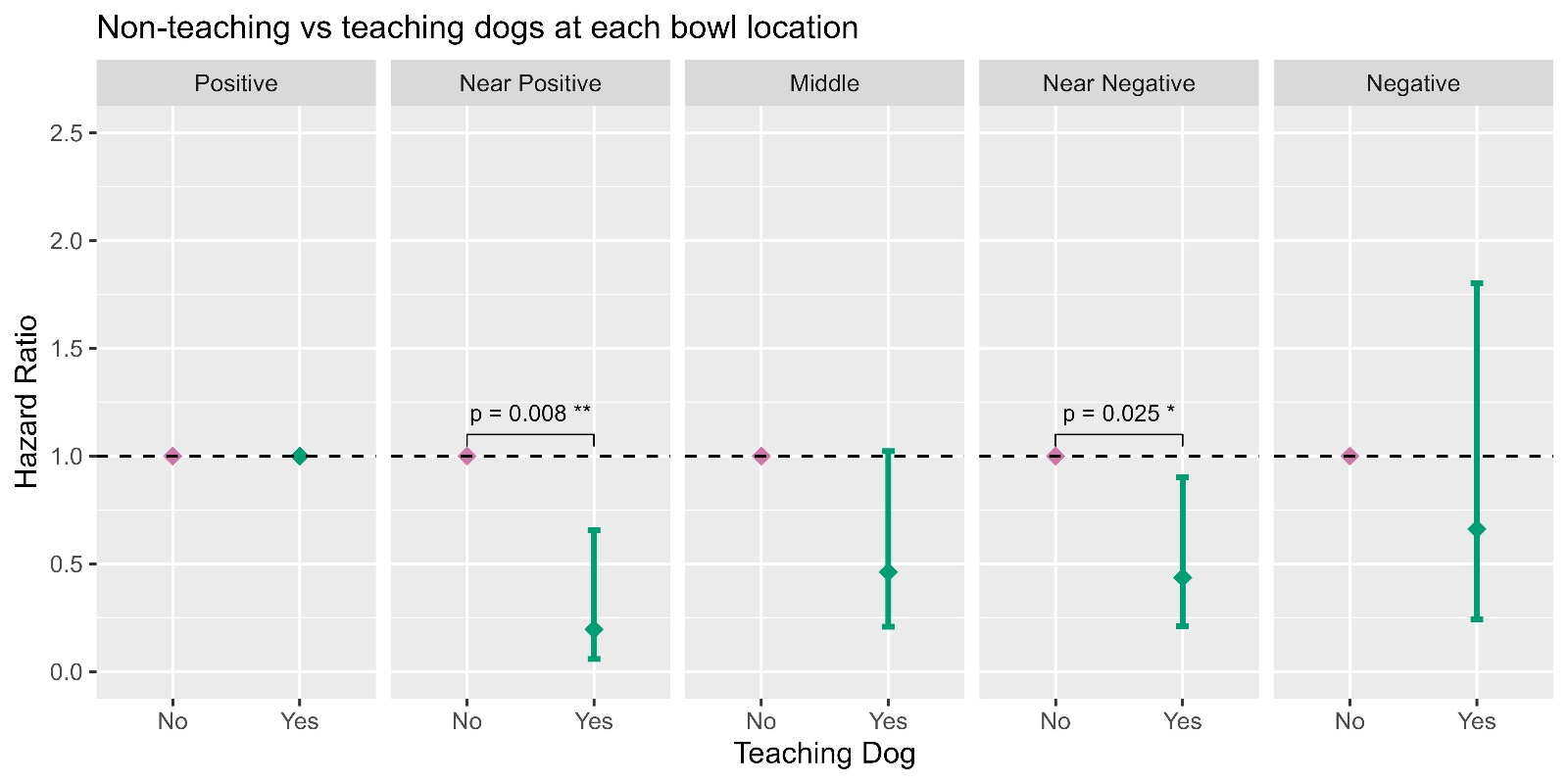


**Supplementary Figure S4.** Plots showing the results of the Cox mixed-effects analysis for the effect of bowl location on the likelihood of approaching for teaching dogs versus non-teaching dogs. The dashed line indicates the reference level (Hazard Ratio = 1): which was set to non-teaching dogs (No), to which registered teaching dogs (Yes) were compared. Error bars represent 95% confidence intervals. Significant differences between teaching and non-teaching dogs are indicated by the square brackets. Significance codes: *** p < 0.001, ** p < 0.01, * p < 0.05.
